# Reconstruction of a Scrotum by Combining Two Skin Flaps in a Ball Shape

**DOI:** 10.1155/2022/2808821

**Published:** 2022-03-19

**Authors:** Kazuya Kashiyama, Motoi Nakano, Akihito Higashi, Shoko Ashizuka, Yuki Moriuchi, Atsuhiko Iwao, Katsumi Tanaka

**Affiliations:** ^1^Department of Plastic and Reconstructive Surgery, Nagasaki Harbor Medical Center, Nagasaki, Japan; ^2^Department of Plastic and Reconstructive Surgery, Nagasaki University Hospital, Nagasaki, Japan

## Abstract

**Background:**

The scrotum functions to maintain spermatogenesis and hormonal production of Leydig cells by preventing the testicles from rising in temperature and protecting them from the outside world. The scrotum, along with the penis, is also an organ that symbolizes masculinity. Therefore, deformity or loss of the scrotum can be a major psychological problem. Various scrotal reconstruction techniques have been reported. In these papers, there is some discussion about the type of skin flap, but little discussion about the method of suturing the skin flap. We devised a way to reconstruct a scrotum to a natural size by suturing two skin flaps together to form a ball shape. *Case Presentation*. Case 1 was a patient with a missing scrotum due to Fournier's gangrene. Total resection of the scrotum, including the bilateral testes, was performed to save his life. Reconstructive surgery was performed 11 days after the initial surgery. Reconstruction was performed using bilateral gluteal fold flaps. Case 2 was a patient with a congenital defect of the scrotum. The testis on the right side exhibited cryptorchidism, and the scrotum was missing, and the testis on the left side was encased in a hypoplastic scrotum. Reconstruction was performed using an internal pudendal artery perforator flap.

**Conclusion:**

There are two types of scrotal defects: those with testes present and those with testes missing. This method can be used for both types of scrotal defects, and we were able to create a scrotum that satisfied each patient.

## 1. Background

The scrotum functions to maintain spermatogenesis and hormonal production of Leydig cells by preventing the testicles from rising in temperature and protecting them from the outside world [1]. The scrotum, along with the penis, is also an organ that symbolizes masculinity. Therefore, deformity or loss of the scrotum can be a major psychological problem. Congenital anomalies, trauma, malignancy, and infections such as Fournier's gangrene can cause scrotal defects [2, 3]. In recent years, scrotum reconstruction has become a very important issue in terms of gender-affirming surgery. Various scrotal reconstruction techniques have been reported [2, 4–6]. In these papers, there is some discussion about the type of skin flap, but little discussion about the method of suturing the skin flap. We devised a way to reconstruct a scrotum with a natural size by suturing two skin flaps together to form a ball shape.

## 2. Case Presentation

### 2.1. Case 1

**Figure 1:**
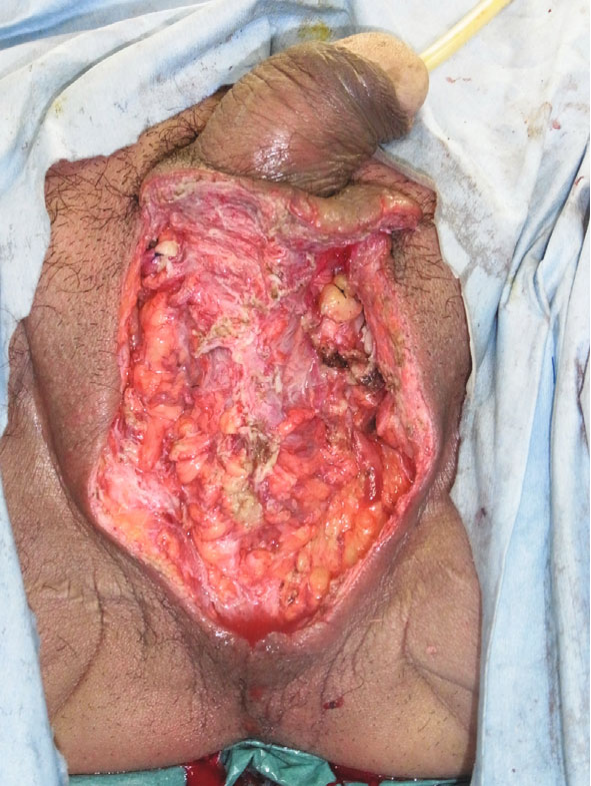
Case 1, primary surgery. Bilateral scrotum and testes were resected.

**Figure 2:**
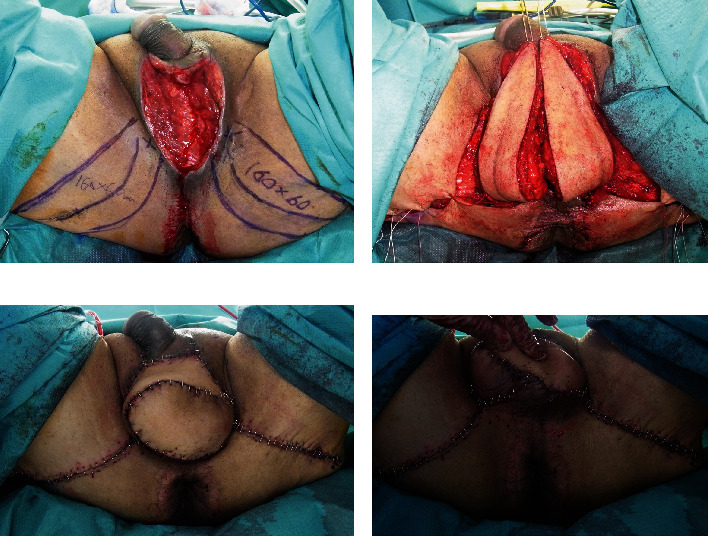
Case 1, reconstruction of a scrotum. (a) Skin flap design. X indicates a perforator. (b) Skin flaps were elevated. (c) Immediately postsurgery anterior view of the scrotum. (d) Immediately postsurgery posterior view of the scrotum.

**Figure 3:**
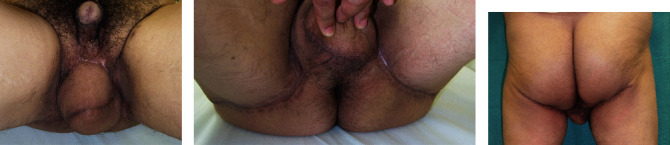
Case 1, eight months after reconstruction surgery. (a) Anterior view of the scrotum. (b) Posterior view of the scrotum. (c) Standing view.

The patient was a 66-year-old Japanese man who had type 2 diabetes. During treatment for epididymitis, the patient developed Fournier's gangrene and was rushed to our hospital. On the same day, a scrotal resection, including the testes, was performed by a urologist (Figure 1). The patient was then referred to the plastic surgery department for scrotal reconstruction. Reconstruction of the scrotum was performed 11 days after the initial surgery. The operation was performed with the patient in the lithotomy position under general anesthesia. Reconstruction was performed using bilateral gluteal fold flaps (Figure 2(a)). The flap was rotated by more than 180 degrees towards the defect in a tension-free manner (Figure 2(b)). Two gluteal fold flaps were combined in a ball shape and sutured together to reconstruct the scrotum (Figures 2(c) and 2(d)). Immediately after the surgery, the patient complained that the reconstructed scrotum was too large and interfered with walking. However, during follow-up, the reconstructed scrotum gradually shrank. Eight months after surgery, there were no problems with the shape or size of the scrotum, and the patient was satisfied with the results (Figures 3(a)–3(c)).

### 2.2. Case 2

**Figure 4:**
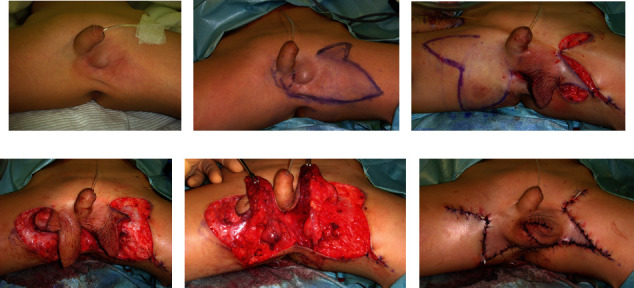
Case 2. (a) Presurgery. (b) Skin flap design of the left side. (c) Skin flap design of the right side. (d) Skin flaps were elevated. (e) Testes are present. (f) Immediately postsurgery.

**Figure 5:**
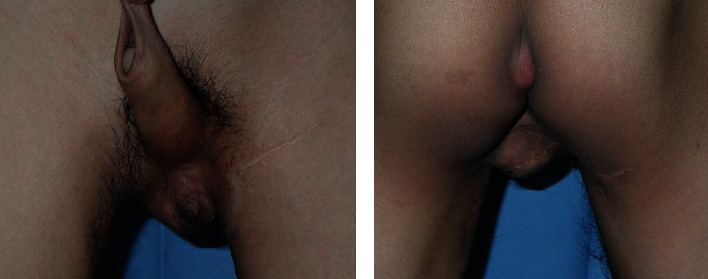
Case 2, eleven years after reconstruction surgery. (a) Anterior view of the scrotum. (b) Posterior view of the scrotum.

The patient was a two-year-Japanese boy who had popliteal pterygium syndrome. The testis on the right side exhibited cryptorchidism, and the scrotum was missing (Figure 4(a)). The testis on the left side was encased in a hypoplastic scrotum. The penis was located slightly to the right of the midline. Reconstruction was performed using an internal pudendal artery perforator flap. Two flaps were combined in a ball shape and sutured together to reconstruct the scrotum. Bilateral testes were wrapped and fixed in the inner envelope space of the two skin flaps (Figures 4(b)–4(e)). At 11 years postoperatively, the testes are still in the reconstructed scrotum. The scrotum is a suitable size and hangs down in the standing position, and the patient was satisfied with the results (Figure 5).

## 3. Discussion and Conclusions

Because the skin of the scrotum is extremely elastic, direct suturing is often possible in cases of partial defects [2]. However, if the defect is large or if there are cosmetic concerns, some form of reconstructive surgery should be considered.

There are two possible forms of total loss of the scrotum. The first is the total loss of the scrotum including the testes. The other is a total loss of the scrotum with the testes and spermatic cord remaining. Malignant tumors and infections may require removal of the scrotum, including the testes and spermatic cord. Total loss of the scrotum due to trauma is often caused by clothing being caught in machinery. In such cases, the testicles and spermatic cord are spared from damage due to the epididymis reflex at the time of injury, and degloving injury often occurs between the internal and external spermatic fascia [7–10].

When considering scrotal reconstruction, two issues need to be considered. One is a cosmetic issue, and the other is a functional issue of testicular protection. Several scrotum reconstruction methods have been reported, including skin grafts, fasciocutaneous flaps, and free flaps, all of which have advantages and disadvantages [1, 11–14].

Skin grafting is a simple method that can be used with or without remaining testes or the spermatic cord and is very useful in terms of testicular cooling because they are covered with a very thin skin [15, 16]. However, reconstruction with skin grafts does not look good [17]. It has also been reported that the presence of the testicles under thin skin prevents patients from leading a normal sex life for fear of hurting them [18, 19]. Therefore, reconstruction with more soft tissue such as a skin flap is preferred if possible [2, 11].

Several types of scrotum reconstruction with skin flaps have been reported, such as gluteal fold island flaps [2], pudendal thigh flaps [20], and gracilis myocutaneous flaps [2, 21–26]. The problem with reconstructing the scrotum with a skin flap is that the flap is bulky due to excessive soft tissue [4]. Therefore, a moderate size and thickness skin flap is needed to create an appropriately sized scrotum. We also have to take into account that the flap shrinks postoperatively due to elasticity [27], and the reconstructed scrotum may be deformed due to contraction of the skin flap.

In cases where the testes or spermatic cord remains, a skin flap large enough to contain the testes or spermatic cord is required. Since the scrotal region is symmetrical, it is often reconstructed using two skin flaps from the left and right sides [2, 4–6, 28, 29]. In this study, we tried to find a way to suture the skin flap so that it would morphologically resemble the scrotum and be able to contain a large space.

In case 1, we used a gluteal fold flap. Although there has been a report of scrotal reconstruction using bilateral gluteal fold flaps in the past, in the reported cases, the two flaps were sutured symmetrically at the midline [2]. We devised a new method for suturing the skin flaps. When suturing, the apex of one skin flap was fixed around the middle of the other skin flap to make a ball with the two skin valves and this was sutured. A baseball ball is made by using two sheets of skin-flap-like cloth, and the same thing was done with skin flaps to create a scrotum. Unlike combining two longitudinal flaps in this method, the force of contraction of the flap works in the direction of tightening the other flap, resulting in an overall reduction in the size of the valve while maintaining its shape. Therefore, the postoperative form is maintained.

In case 2, we used an internal pudendal artery perforator flap [30]. Skin flaps were created from the left and right sides in order to adequately cover the testes on both sides. Eleven years after the surgery, hair growth was observed in the scrotum reconstructed by the skin flap. In prepubertal patients, careful consideration should be given to the choice of flap harvest site.

If the testes remain, it is necessary to obtain sufficient space to contain them. A single adult testis weighs about 15 g, is 5 cm long, 3 cm wide, and 1.5-2 cm thick, with a volume of about 20 cc [31–33]. There is some discussion about the type of skin flap used in the literature, but little discussion about the method of suturing the skin flap. It is thought that if the skin flap is sutured in a ball-like combination, it is possible to obtain an appropriate internal space rather than if the same size skin valves were sutured vertically. In addition, unlike combining two longitudinal skin flaps, when skin flaps are combined using this method, the force of contraction of the skin flaps works in the direction of tightening each other, resulting in an overall reduction in the size of the skin flaps while maintaining their shape. Therefore, the postoperative morphology can be maintained. The problem is that the concept of creating an appropriate space inside by combining skin flaps may create a dead space. Therefore, adequate drainage should be performed when suturing the skin flap using this approach.

## Data Availability

Records and data pertaining to this case are in the patients' secure medical records at the Nagasaki Harbor Medical Center. All data researched by literature review are included in this paper.
